# Endothelial–Mesenchymal Transition and Possible Role of Cytokines in Streptozotocin-Induced Diabetic Heart

**DOI:** 10.3390/biomedicines13051148

**Published:** 2025-05-09

**Authors:** Hsu Lin Kang, Ákos Várkonyi, Ákos Csonka, András Szász, Tamás Várkonyi, Anikó Pósa, Krisztina Kupai

**Affiliations:** 1 Department of Oral Biology and Experimental Dental Research, Faculty of Dentistry, University of Szeged, 6703 Szeged, Hungaryszenzor.varkonyiakos@gmail.com (Á.V.); szasz.andras@szte.hu (A.S.); aniko.posa@icloud.com (A.P.); 2 Department of Internal Medicine, Albert Szent-Györgyi Medical School, University of Szeged, 6703 Szeged, Hungary; csonka.akos81@gmail.com (Á.C.); varkonyi.tamas@med.u-szeged.hu (T.V.)

**Keywords:** EndMT, STZ, Diabetic Heart

## Abstract

**Background:** Although endothelial mesenchymal transition (EndMT) has been characterized as a basic process in embryogenesis, EndMT is the mechanism that accelerates the development of cardiovascular diseases, including heart failure, aging, and complications of diabetes or hypertension as well. Endothelial cells lose their distinct markers and take on a mesenchymal phenotype during EndMT, expressing distinct products. **Methods:** In this study, type 1 Diabetes mellitus (T1DM) was induced in rats with streptozotocin (STZ) by intraperitoneal injection at a 60 mg/kg dose. Diabetic rats were randomly divided into two groups, namely, control and diabetic rats, for 4 weeks. Heart, aorta, and plasma samples were collected at the end of 4 weeks. Sequentially, biochemical parameters, cytokines, reactive oxygen species (ROS), protein expression of EndMT markers (Chemokine C-X-C motif ligand-1 (CXCL-1), vimentin, citrullinated histone H3 (H3Cit), α-smooth muscle actin (α-SMA), and transforming growth factor beta (TGF-β) and versican), components of the extracellular matrix (matrix metalloproteinase 2 (MMP-2), tissue inhibitor of metalloproteinase-1(TIMP-1), and discoidin domain tyrosine kinase receptor 2 (DDR-2)) were detected by ELISA or Western blot, respectively. **Results:** Cytokines and ROS were increased in diabetic hearts, which induced partial EndMT. Among EndMT markers, histone citrullination, α-SMA, and CXCL-1 were increased; vimentin was decreased in DM. The endothelial marker endothelin-1 was significantly higher in the aortas of DM rats. Interestingly, TGF-β showed a significant decrease in the diabetic heart, plasma, and aorta. Additionally, MMP-2/TIMP-1 levels also decreased in DM. **Conclusions:** To sum up, the identification of molecules and regulatory pathways involved in EndMT provided novel therapeutic approaches for cardiac pathophysiological conditions.

## 1. Introduction

Diabetes mellitus (DM) is a leading global health burden and a major risk factor for cardiovascular disease (CVD). Despite intensive glucose-lowering strategies, patients with diabetes continue to experience elevated cardiovascular morbidity and mortality, pointing to underlying mechanisms beyond hyperglycemia alone [1,2,3]. A growing body of evidence identifies endothelial-to-mesenchymal transition (EndMT) as a crucial pathophysiological process linking diabetes to cardiac dysfunction and vascular complications. EndMT refers to the transdifferentiation of endothelial cells (ECs) into mesenchymal-like cells. During this transition, ECs lose their characteristic markers, such as VE–cadherin and CD31, and acquire mesenchymal features, including α-SMA and collagen I. This shift promotes extracellular matrix (ECM) deposition, fibrosis, and vascular stiffness. In the diabetic context, hyperglycemia, oxidative stress, and chronic inflammation act synergistically to drive EndMT both locally and systemically. In vitro studies using human umbilical vein endothelial cells (HUVECs) show that high glucose environments induce EndMT through the TGF-β1/ERK pathway. Suppression of this transition has been achieved with ERK inhibitors like PD98059 or antioxidants such as N-acetylcysteine [4,5,6]. Consistent with these findings, cardiac tissues from diabetic patients display increased mesenchymal marker expression, indicating persistent EndMT even under controlled glycemia. Non-coding RNAs (e.g., miR-27a-3p, miR-200c) and long non-coding RNAs also regulate EndMT via epigenetic modifications, including DNA methylation and histone acetylation [7,8]. This epigenetic remodeling supports the concept of metabolic memory, where prior hyperglycemia causes lasting endothelial changes, even after normalization of blood glucose [9,10,11]. Animal models, particularly streptozotocin (STZ)-induced diabetic rats, recapitulate the fibrotic cardiac phenotype observed in diabetic humans. These rats display both systolic and diastolic dysfunction, coupled with elevated reactive oxygen species (ROS), inflammatory signaling, and EndMT activation [12]. Elevated levels of TNFRSF21, a member of the tumor necrosis factor receptor superfamily, have been shown to mediate EndMT under diabetic conditions and correlate with impaired cardiac function [13]. The diabetic myocardium exhibits chronic inflammation and increased immune cell infiltration. Elevated levels of cytokines such as IL-1β, IL-6, and TGF-β, as well as activation of NF-κB, suggest a proinflammatory environment that contributes to EndMT induction [14,15]. ROS not only damage cellular structures directly but also activate pathways like JNK, p38-MAPK, and PKC, amplifying inflammation and cellular transformation [16]. This creates a feedforward loop in which oxidative stress and inflammation mutually reinforce EndMT progression. Recent findings also point to the role of extracellular vesicles (EVs) as mediators of EndMT. EVs derived from mesenchymal stem cells (MSCs) of diabetic animals can induce EndMT and impair angiogenesis in recipient endothelial cells. Conversely, EVs from non-diabetic donors have shown restorative effects [17]. From a developmental perspective, EndMT is essential in embryonic heart valve formation. However, in the diabetic heart, this process is aberrantly reactivated, leading to pathological fibrosis. Endothelial cells exposed to high glucose and proinflammatory stimuli acquire mesenchymal characteristics and contribute to excess ECM production and stiffness of the vascular wall [18,19]. Dysregulation of MMP-2, MMP-9, and their tissue inhibitors further exacerbates ECM accumulation and fibrotic remodeling [20].

We hypothesize that an imbalance in ROS production and cytokine signaling promotes sustained inflammation and EndMT activation in the diabetic heart. The breakdown in matrix regulation and mesenchymal activation plays a central role in the structural and functional decline seen in diabetic cardiomyopathy. Targeting the molecular pathways underlying EndMT—including TGF-β signaling, oxidative stress response, and epigenetic regulation—may offer new therapeutic avenues to slow or reverse diabetes-induced cardiovascular deterioration. Understanding and modulating EndMT could thus shift the paradigm in diabetic heart disease management.

## 2. Materials and Methods

### 2.1. STZ Injection

Male Wistar rats from BRC in Szeged, Hungary, were housed in cages as the experimental animals. Before they were sacrificed, the habitat was maintained for six weeks at a consistent temperature of 20–22 °C and humidity of 40–50% with a 12/12 h light/dark cycle. The rats weighed between 200 and 230 g, and they were fed and hydrated without restriction. Weighted measurements of body weight were taken both at the beginning and the end of this study. We just needed the bare minimum of rats to perform this experiment, and the required sample size was established using the resource equation and our prior trial as a guide (*n* = 5–15, depending on assay). We also piloted this trial in our laboratory. Every procedure was approved by the European Community rules and complied with the Directive of the European Parliament (2010/63EU). (Ethics license, University of Szeged: XXXIX./2040/2023). We were able to distinguish randomly between the Type 1 Diabetes Mellitus (T1DM) group and the control group following acclimatization. Intraperitoneal (IP) injections of saline were given to the control group, and 60 mg/kg/body weight of STZ was given to the T1DM group. Throughout the trial, the weight of each rat was noted, and, at the fourth week, they were all sacrificed. Each rat’s heart and aorta were carefully removed and used. Blood samples were collected as well, for future measurements. Before analysis, every tissue was kept in −80 °C for biological measures [12,21].

### 2.2. Determination of Cardiac Cytokines ROS, EndMT Markers (IL-18,6,33,10, lL-β1, ROS, Peroxynitrite (ONOO^−^), Nitric Oxide Synthases (NOS) Isoforms, and TNF-α Concentrations

Cardiac samples were homogenized in ice-cold phosphate buffer (pH 7.4) for 20 s. After centrifugation (10 min, 3500 rpm, 4 °C), supernatants were collected carefully and used for ELISA and protein measurements. According to the manufacturer’s datasheet (Gen Asia Biotech Co., Ltd., Shanghai, China), the amount of either 40 µL cardiac tissue supernatant or 50 µL standard solution was added to the wells that were pre-coated with cytokine/ROS monoclonal antibody. Additionally, the supernatant-containing wells were completed with a second antibody labeled with biotin. Subsequently, Streptavidin-HRP was also added to both the supernatant and the standard-containing wells, which consequently formed an immune complex with a biotin-labeled antibody. After the incubation procedure at 37 °C, the plate was washed five times; thus, the unbound enzymes have been removed. For the color development, substrate solutions A and B were added to the wells and incubated for 10 min at 37 °C hidden from light. As the last step, the stop solution was pipetted, which resulted in a change from blue to yellow with the effect of acid. The optical density was measured at 450 nm. According to standard concentrations and the corresponding OD values, the linear regression equation of the standard curve and the samples’ concentrations were calculated.

### 2.3. Matrix Metalloproteinase 2 (MMP-2), MMP-2 Zymography

The procedure for performing gelatin zymography was carried out as previously mentioned [22]. From rat heart homogenates, MMP-2 was extracted as follows: on an 8% SDS-polyacrylamide gel copolymerized with 2 mg/mL gelatin from pig skin, 50 μg protein/lane was loaded and separated by electrophoresis under non-reducing conditions (Sigma-Aldrich; St. Louis, MO, USA). Following electrophoresis, gels were gently shaken and cleaned in 2.5% Triton-X 100 before being incubated for 20 h at 37 °C in a zymography development buffer (50 mM Tris-HCl, pH 7.5, containing 200 mM NaCl and 5 mM CaCl_2_). After destaining and staining zymographic gels with a 0.05% Coomassie Brilliant Blue R-250 solution, zymograms were scanned, and density was calculated by ImageJ software version 1.52.

### 2.4. Western Blot Analyses of Cardiac Discoidin Domain Tyrosine Kinase Receptor 2 (DDR-2), Citrullinated Histone H3 (H3Cit), Vimentin

To determine the molecular mass of DDR-2, citrullinated histone and vimentin SDS-PAGE was performed using 10% (*w*/*v*) acrylamide gel. The proteins were diluted (to 20 ug) in Laemli sample buffer (Invitrogen- Waltham, MA, USA). Following SDS-PAGE, the proteins were transferred onto a nitrocellulose membrane (Invitrogen Waltham, United States) and blocked in TBS-Tween buffer (20 mM Tris (pH 7.4–7.6), 0.5 M NaCl, 0.05% Tween) with 5% (*w*/*v*) milk. The membranes were then incubated overnight in TBST ween and 1% milk in the presence of primary antibody at 4 °C. The next day, the membranes were incubated with secondary horseradish peroxidase, and detection was carried out using enhanced chemiluminescence (Amersham Biosciences, Buckinghamshire, UK) [23].

### 2.5. Determination of Cardiac Myeloperoxidase (MPO) Activity

Hexadecyltrimethylammonium bromide (0.5%) was added to the phosphate buffer (pH 6.0) before the heart tissues were homogenized. The homogenized samples were cooled to 37 °C in a water bath, and then they were placed in liquid nitrogen. After completing these procedures three times, the samples were centrifuged at 15,000× *g* for 15 min at 4 °C, and the supernatants were gathered. The measurement was carried out using a plate with 96 wells. A total of 280 µL of o-dianisidine diHCL and 12 µL of either the sample or the standard (diluted from peroxidase) were pipetted into the plate. After shaking for 59 s, cardiac MPO activity was measured at 490 nm and expressed as µU/mg protein.

### 2.6. Protein Content Measurement

Using a commercial protein assay kit (Bio-Rad Labs, Hercules, CA, USA), aliquots (20 μL) of the diluted samples were mixed with 980 μL of distilled water, with 300 μL of BCA reagent added to each sample. After mixing and following a 10-min incubation, the samples were assayed spectrophotometrically at 595 nm. The protein level was expressed as ug protein/uL.

### 2.7. Statistical Analysis

All data are expressed as means + SEM. Differences between the two groups were determined using an unpaired Student’s *t*-test. A value of *p* < 0.05 was considered statistically significant.

## 3. Results

### 3.1. The Expression of Various Cytokines in the Heart

**Figure 1:**
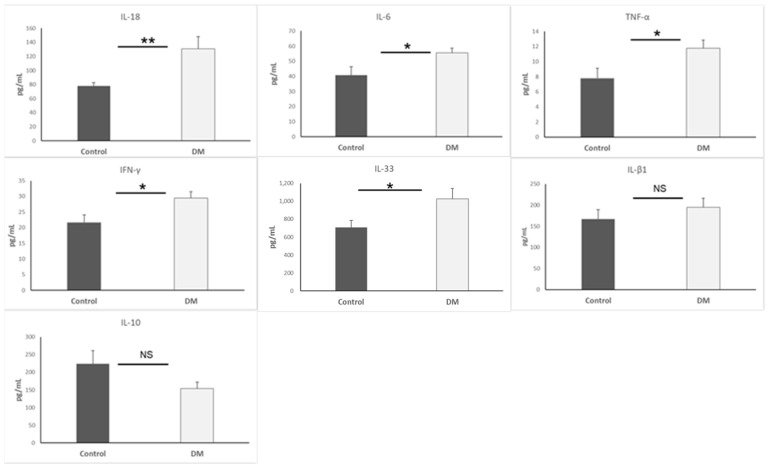
Cytokine expression in heart. *, *p* < 0.05; **, *p* < 0.01; NS, non-significant.

In this study, cardiac cytokines were measured to map the inflammatory status in the heart. We obtained significant results (IL-6, IL-33, IFN-γ, and TNF-α: *p* < 0.05, IL-18: *p* < 0.01) for primary confirmation. The inflamed status of the heart was determined due to a high level of inflammatory cytokines in the STZ-DM group. Results are presented as mean + SEM, n = 5–9/group (Figure 1). 

### 3.2. Basic ROS Examination

**Figure 2:**
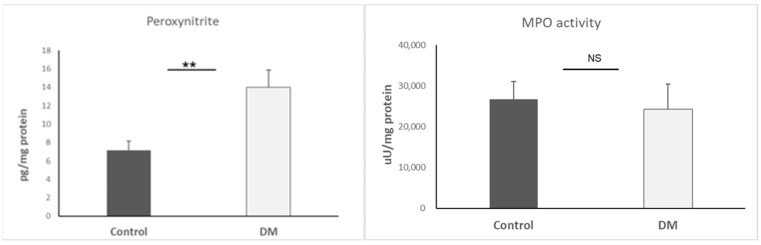
Peroxynitrite and MPO activity expression in heart. **, *p* < 0.01; NS, non-significant.

ROS is one of the main root causes of an EndMT microenvironment. We utilized Peroxynitrite (ONOO^−^) and MPO to estimate the ROS condition. The data only presented increased peroxynitrite (*p* < 0.01)) in STZ, suggesting a high ROS surrounding in the cardiac area. Results are presented as mean + SEM; n = 4–9/group (Figure 2). 

### 3.3. Nitric Oxide Synthases (NOS) Determination

**Figure 3:**
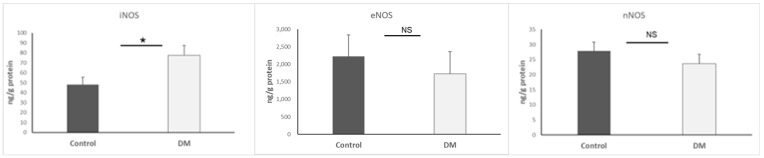
iNOS, eNOS, and nNOS expression in heart. *, *p* < 0.05; NS, non-significant.

Here, we detected inducible NOS (iNOS), endothelial NOS (eNOS), and neuronal ROS (nROS) in cardiac tissue. Our data showed that iNOS was only significantly higher in the STZ-DM group (*p* < 0.05). As a result, immune cells were likely to accumulate in the cardiovascular system. Results are presented as mean + SEM; n = 5–6/group (Figure 3). 

### 3.4. The Assessment of EndMT by DDR-2, MMP-2, and TIMP-1

**Figure 4:**
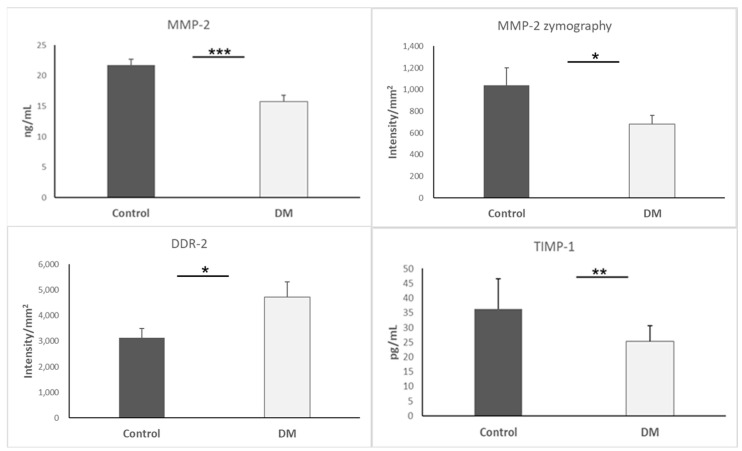
MMP-2, MMP-2 zymography, DDR-2, and TIMP-1 expression in heart. *, *p* < 0.05; **, *p* < 0.01; *** *p* < 0.001.

For the purpose of assessing EndMT, we observed DDR-2, MMP-2 activity, and TIMP-1. The underlying mechanism is that TIMP-1 is capable of inhibiting MMP-2, and DDR-2 can promote MMP-2-mediated proliferation. Interestingly, our data indicated that MMP-2 (*p* < 0.001) and TIMP-1 (*p* < 0.01) were decreased simultaneously in the hearts of the STZ-DM group. Nevertheless, DDR-2 was increased in the hearts of the STZ-DM group (*p* < 0.05). Results are presented as mean + SEM; n = 5–8/group (Figure 4). 

### 3.5. Biomarkers of Mesenchymal Cells and Neutrophils

**Figure 5:**
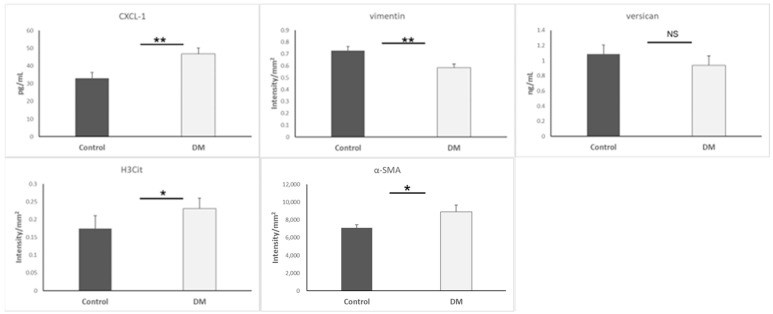
CXCL-1, Vimentin, H3Cit, α-SMA and versican expression in heart. *, *p* < 0.05; **, *p* < 0.01; NS, non-significant.

Continuously, we measured citrullinated histone to confirm whether CXCL1 attracted neutrophils. Additionally, we measured some typical biomarkers (vimentin, versican, and α-SMA) to sense mesenchymal cells. Our data indicated that vimentin was lower (*p* < 0.01) but α-SMA was higher (*p* < 0.05) in the STZ-DM group. Additionally, the STZ-DM group presented high CXCL1 (*p* < 0.01) and H3Cit (*p* < 0.05) levels, proving that neutrophils were able to infiltrate the heart during EndMT. Results are presented as mean + SEM; n = 6–15/group (Figure 5). 

### 3.6. TGF-β Level of Heart, Aorta, and Plasma

**Figure 6:**
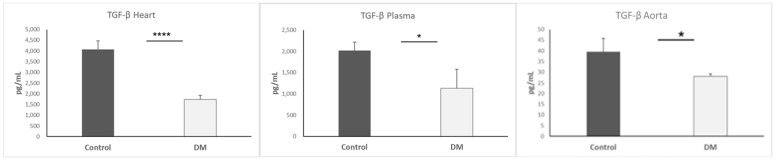
TGF-β expression in heart, plasma, and aorta. *, *p* < 0.05; **** *p* < 0.0001.

TGF-β is an important factor for EndMT and an upstream indicator to trigger EndMT. We continued to detect TGF-β expression since we obtained lower vimentin in the STZ-DM group. The TGF-β level in the heart of the STZ-DM group was exceedingly lower, as proven by statistical significance (*p* < 0.0001), as well as in the aorta (*p* < 0.05) and plasma (*p* < 0.05). Results are presented as mean + SEM; n = 4–8/group (Figure 6). 

### 3.7. Endothelin-1 Expression in Heart and Aorta

**Figure 7:**
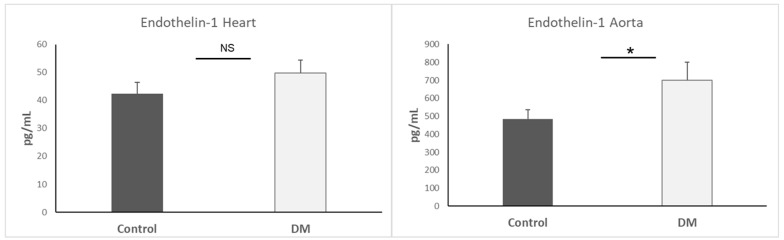
Endothelin-1 expression in heart and aorta. *, *p* < 0.05; NS, non-significant.

Endothelin-1 has been linked to the pathophysiology of other biological diseases, especially irregular EndMT [24]. We also measured endothelin-1 expression in the heart and aorta. Our data only indicated that endothelin-1 expression in the aorta of the STZ-DM group was higher than the control group (*p* < 0.05). Results are presented as mean + SEM; n = 5–7/group (Figure 7). 

## 4. Discussion

Our study provides an integrative view of how diabetic conditions promote EndMT and fibrotic remodeling in the heart, highlighting several intersecting molecular pathways. Using the STZ-induced rat model of T1DM, which replicates key human pathological features such as chronic hyperglycemia, systemic inflammation, and cardiac remodeling, we identified a multifaceted interplay among proinflammatory cytokines, oxidative stress, extracellular matrix dysregulation, and epigenetic modifications contributing to endothelial dysfunction and EndMT. These findings offer translational insights relevant to diabetic cardiovascular complications in humans. A central feature of diabetic pathology is the systemic proinflammatory state, which we confirmed by elevated levels of IL-6, IL-18, IL-33, TNF-α, and IFN-γ in cardiac tissue. These cytokines are well-documented contributors to cardiac inflammation and have previously been implicated in diabetic cardiomyopathy [14,25]. Notably, TNF-α may act upstream to activate CXCL1 signaling [26], which we found to be upregulated, suggesting a potential biomarker and effector of EndMT. Prior studies have associated CXCL1 and CXCL2 with neutrophil infiltration and myocardial injury in diabetes [27,28,29,30,31]; our results expand on this by connecting CXCL1 expression to EndMT regulation, a link that remains relatively underexplored. Our data also reinforce the pivotal role of oxidative stress in diabetic heart disease. Elevated levels of ROS and iNOS were observed, consistent with prior studies demonstrating ROS-induced endothelial injury [16,32,33,34]. In particular, the accumulation of peroxynitrite (ONOO^−^) mirrors findings from ischemia-reperfusion models [33], further implicating oxidative damage in the cardiac remodeling seen in diabetes. This oxidative stress environment appears to disrupt extracellular matrix homeostasis. We observed significantly reduced MMP-2 activity and TIMP-1 expression, aligning with evidence that oxidative stress inhibits MMP function at high ROS levels [35,36,37,38]. These changes are consistent with a profibrotic shift that facilitates EndMT and tissue remodeling [39,40]. Interestingly, versican—a known ECM proteoglycan and EndMT marker—was not altered in our model, suggesting temporal or disease-stage-specific regulation [41]. An unexpected finding was the reduced TGF-β expression in both the heart and aorta of diabetic rats. While TGF-β is a canonical inducer of EndMT and fibrosis, its downregulation in our model contrasts with reports of increased TGF-β activity in other cardiovascular disease contexts [18,19,42,43,44]. This suggests a complex, possibly compensatory, regulation of TGF-β signaling in early versus advanced stages of diabetic cardiovascular disease. Supporting this, prior studies show that TGF-β inhibition can exacerbate aortic wall inflammation in animal models [45]. We also found reduced vimentin content, which may have dual consequences: impairing cytoskeletal integrity and removing its inhibitory effect on ROS synthesis, thereby amplifying oxidative stress [46,47,48,49,50,51]. This could also explain the observed increase in H3Cit, an epigenetic marker of inflammation linked to neutrophil extracellular trap (NET) formation [52,53,54]. The relationship between vimentin loss, increased ROS, and H3Cit provides a novel mechanistic insight into how epigenetic and cytoskeletal changes may jointly promote inflammation and EndMT.

Further, our study confirms increased expression of α-SMA, a hallmark of mesenchymal transformation. This aligns with in vitro findings of high-glucose-induced EndMT in human endothelial cells [4,55] and reinforces the relevance of our in vivo results. The upregulation of DDR2—a collagen-binding receptor sensitive to matrix stiffness and hyperglycemia—adds another layer of complexity. DDR2 has recently been implicated in enhancing EndMT in fibrotic environments [56,57,58], and our findings suggest it may act as a mediator between mechanical and metabolic stress in diabetic cardiac remodeling (Table 1).

Recent research also highlights novel mediators of EndMT. Kallikrein-related peptidase 8 (KLK8), for instance, was found to promote EndMT through VE–cadherin cleavage and enhanced TGF-β1 signaling, a mechanism not previously explored in diabetic hearts [59]. Additionally, epigenetic regulators, like miR-126-3p (Table 2), have shown potential in modifying EndMT outcomes in diabetic models [7]. Our findings of increased H3Cit are particularly significant in this context, as recent studies show histone citrullination is modulated by oxidative stress and linked with NET formation and fibrotic remodeling [60]. Furthermore, confirmation of EndMT markers in human diabetic heart tissues validates the translational relevance of our animal model [61].

**Figure 8:**
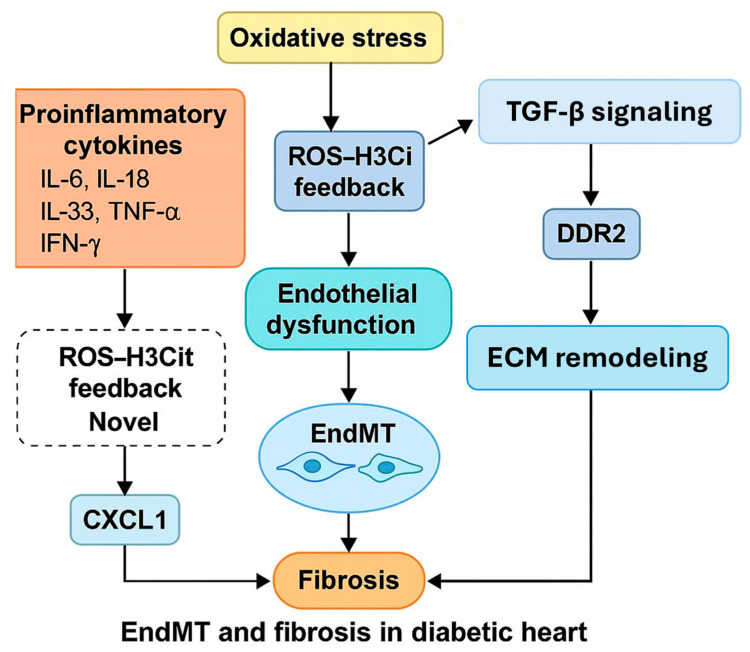
Brief summary of EndMT pathway in the diabetic heart of T1DM. Dotted box, another assumed pathway results in fibrosis.

In summary (Figure 8), our integrative analysis identifies multiple converging pathways that mediate EndMT and fibrosis in the diabetic heart. We propose a model in which hyperglycemia-induced oxidative stress and inflammation initiate endothelial dysfunction, while chemokine signaling, ECM remodeling, and epigenetic changes sustain and amplify this response. The interplay between reduced TGF-β signaling, increased DDR2 expression, and ROS-H3Cit feedback loops is particularly novel and warrants further exploration. By contextualizing these findings within the broader landscape of diabetic cardiovascular research, we highlight new potential targets for early intervention in diabetic cardiomyopathy.

### Table 1 and Table 2. Key Molecular Regulators and Therapeutic Targets Related to EndMT in Diabetic Cardiomyopathy

The two tables summarize established and novel molecular factors associated with EndMT in the context of streptozotocin-induced diabetes. It includes experimental findings from rat models and recent insights from 2023 to 2024 studies, highlighting microRNAs, signaling molecules, and therapeutic agents. Understanding these targets may provide new avenues for preventing cardiac fibrosis and vascular complications in diabetes.

## 5. Conclusions

**Figure 9:**
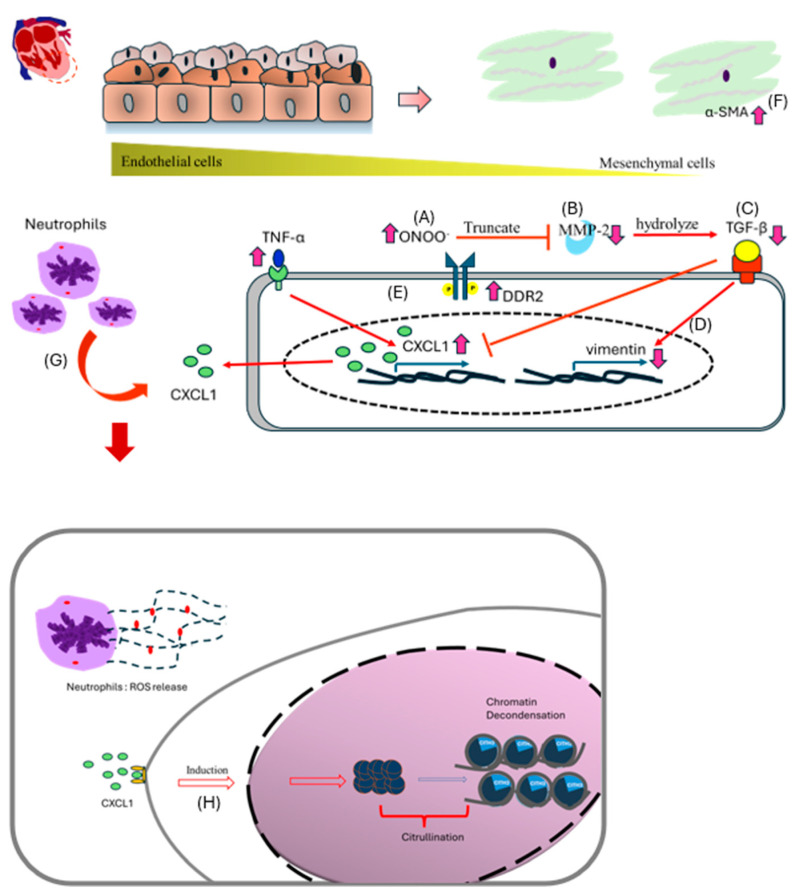
Scheme showing possible EndMT in the heart by STZ-induced T1DM. (**A**) In STZ DM rats, increased peroxynitrite truncates MMP-2, rendering it inactive. (**B**,**C**) MMP-2 facilitates the hydrolysis of TGF-β, causing activated TGF-β to induce the downstream pathway. In STZ DM, decreased MMP-2 suppressed TGF-β transduction. (**D**) Vimentin is one biomarker in mesenchymal cells during EndMT that is activated by TGF-β; a decrease in TGF-β causes a corresponding drop in vimentin. (**E**) On the other side, elevated TNF-α led to an increase in CXCL1. DDR2, a different biomarker during EndMT, was aberrantly elevated in the interim. Certain papers about in situ carcinoma suggest that diabetic heart disease may also have elevated DDR2. (**F**) In mesenchymal cells, higher α-SMA is a classical phenomenon during EndMT. (**G**) CXCL1 from endothelial cells is likely to attract immune cells, particularly neutrophils. In the diabetic heart, neutrophils may close as a result of an inflammatory signal being drawn to them. (**H**) CXCL-1 may induce citrullination in neutrophils.

In conclusion, our study highlights the significant role of inflammatory pathways, oxidative stress, and extracellular matrix remodeling in the progression of EndMT within the diabetic heart and aorta. The elevated expression of proinflammatory cytokines (e.g., TNF-α, IL-6) and chemokines, such as CXCL1, together with increased reactive oxygen species (ROS), suggests a synergistic inflammatory microenvironment in STZ-induced diabetic rats. Concurrently, a reduction in MMP-2 and TIMP-1 activity, alongside altered expression of EndMT markers such as α-SMA and DDR2, indicates active matrix reorganization and endothelial transdifferentiation. Notably, the decrease in TGF-β and vimentin expression underscores potential dysregulation of protective mechanisms, possibly contributing to cardiac fibrosis and dysfunction. The observed histone H3 citrullination further implies a mechanistic link between epigenetic modification and oxidative-inflammatory signaling. These findings collectively emphasize that EndMT is not merely a secondary phenomenon but a potential driving force in diabetic cardiomyopathy. Targeting these interconnected pathways may offer new avenues for therapeutic intervention in diabetes-associated cardiac remodeling (Figure 9).

## Figures and Tables

**Table 1:** EndMT and cardiovascular markers in diabetic heart. ↑, Higher expression in STZ-DM model; ↓, Lower expression in STZ-DM model; ≈, non-significant between STZ-DM model and control.

Factor/Marker	Change in STZ-DM Model	Role in EndMT/Cardiovascular Pathophysiology	Key References
TGF-β	↓	Master EndMT inducer; low levels impair mesenchymal transition	[42,43,44]
CXCL-1	↑	Chemokine attracting neutrophils; pro-inflammatory	[27,28,29,30,31]
TNF-α	↑	Inflammatory cytokine; upstream activator of CXCL-1	[26]
IL-6	↑	Pro-inflammatory; promotes endothelial dysfunction	[14,25]
IL-18	↑	Inflammatory mediator; promotes cytokine storm	[14]
IL-33	↑	Involved in innate immunity and inflammation	[14]
IFN-γ	↑	Stimulates Th1 responses and immune cell activation	[14]
Vimentin	↓	Mesenchymal marker; lower levels suggest impaired transition	[46,47,48,49,50,51]
α-SMA	↑	Mesenchymal marker: elevation indicates EndMT progression	[4,55]
Versican	≈	ECM proteoglycan; no significant change observed	[41]
DDR-2	↑	Collagen receptor; increased in stiff/fibrotic matrix	[56,57,58]
MMP-2	↓	Degrades ECM; decreased activity leads to fibrosis	[35,36,37,38]
TIMP-1	↓	Inhibits MMPs; decreased expression disrupts ECM regulation	[39,40]
H3Cit	↑	Histone modification; indicates neutrophil activation and chromatin remodeling	[52,53,54]
Endothelin-1	↑	Vasoconstrictor; elevated in diabetic aorta, promotes EndMT	[24]
Peroxynitrite	↑	ROS indicator; initiates oxidative damage and EndMT	[33]
iNOS	↑	Enzyme producing NO; high levels promote inflammation	[16,32,33,34]

**Table 2:** Novel EndMT-related markers and therapeutic targets. ↑, Higher expression in STZ-DM model; ↓, Lower expression in STZ-DM model; ≈, non-significant between STZ-DM model and control.

Factor/Marker	Change in STZ-DM Model	Role in EndMT/Cardiovascular Pathophysiology	Key References
miR-200b	↑	Regulates EndMT via TGF-β/Smad pathway; inhibition reduces cardiac fibrosis	[62]
miR-181b	↑	Modulates TGF-β-induced EndMT via targeting Semaphorin 3A; implicated in atrial fibrillation	[63]
miR-200c-3p	↑	Promotes EndMT and intimal hyperplasia in graft vessels	[64]
miR-126-3p	≈	Dynamically regulated in EndMT; involved in fibrosis-related processes	[65]
MFGE8	↓	Suppression promotes EndMT via Smad2/3-Snail signaling	[66]
Serelaxin	Therapeutic	Inhibits EndMT via RXFP1 receptor; reduces fibrosis	[67]
Ghrelin	Therapeutic	Inhibits EndMT; reduces cardiac fibrosis post-MI	[68]
EGCG	Therapeutic	Suppresses EndMT; improves cardiac function	[69]
Liraglutide	Therapeutic	Suppresses EndMT; reduces neointima formation in diabetic mice	[70]
BRD4	↑	Its inhibition reduces EndMT and cardiac fibrosis	[71]

## Data Availability

Data will be made available upon reasonable request to the coauthors.
